# Role of γ-glutamyltranspeptidase in the pathogenesis of *Helicobacter suis* and *Helicobacter pylori* infections

**DOI:** 10.1186/s13567-015-0163-6

**Published:** 2015-03-13

**Authors:** Guangzhi Zhang, Richard Ducatelle, Ellen De Bruyne, Myrthe Joosten, Iris Bosschem, Annemieke Smet, Freddy Haesebrouck, Bram Flahou

**Affiliations:** Department of Pathology, Bacteriology and Avian Diseases, Faculty of Veterinary Medicine, Ghent University, 9820 Merelbeke, Belgium

## Abstract

*Helicobacter* (*H.*) *suis* can colonize the stomach of pigs as well as humans, causing chronic gastritis and other gastric pathological changes including gastric ulceration and mucosa-associated lymphoid tissue (MALT) lymphoma. Recently, a virulence factor of *H. suis*, γ-glutamyl transpeptidase (GGT), has been demonstrated to play an important role in the induction of human gastric epithelial cell death and modulation of lymphocyte proliferation depending on glutamine and glutathione catabolism. In the present study, the relevance of GGT in the pathogenesis of *H. suis* infection was studied in mouse and Mongolian gerbil models. In addition, the relative importance of *H. suis* GGT was compared with that of the *H. pylori* GGT. A significant and different contribution of the GGT of *H. suis* and *H. pylori* was seen in terms of bacterial colonization, inflammation and the evoked immune response. In contrast to *H. pylori*Δ*ggt* strains, *H. suis*Δ*ggt* strains were capable of colonizing the stomach at levels comparable to WT strains, although they induced significantly less overall gastric inflammation in mice. This was characterized by lower numbers of T and B cells, and a lower level of epithelial cell proliferation. In general, compared to WT strain infection, *ggt* mutant strains of *H. suis* triggered lower levels of Th1 and Th17 signature cytokine expression. A pronounced upregulation of B-lymphocyte chemoattractant CXCL13 was observed, both in animals infected with WT and *ggt* mutant strains of *H. suis*. Interestingly, *H. suis* GGT was shown to affect the glutamine metabolism of gastric epithelium through downregulation of the glutamine transporter ASCT2.

**Electronic supplementary material:**

The online version of this article (doi:10.1186/s13567-015-0163-6) contains supplementary material, which is available to authorized users.

## Introduction

*Helicobacter* (*H.*) *pylori* is a Gram-negative bacterium that colonizes the stomach of more than half of the world’s population. Infection with this bacterium can cause gastritis, peptic ulcer disease, gastric adenocarcinoma and mucosa-associated lymphoid tissue (MALT) lymphoma [1-3]. Besides *H. pylori*, non*-H. pylori* helicobacters (NHPH) have also been detected in the stomach of humans and these bacteria cause similar gastric diseases. The risk of developing gastric MALT lymphoma is higher during NHPH infection compared to infection with *H. pylori* [4-9]. *H. suis* is the most prevalent gastric NHPH in humans. Pigs are the natural host of this bacterium, with prevalences reaching 90% or more [10] and most likely, pigs and possibly also pork are the main sources of human *H. suis* infection [4,11-13].

*H. suis* infection seems to persist for life, at least in pigs and rodents used as models for human infections [14]. In pigs, infection causes development of gastritis and a decrease in body weight gain. Moreover, the bacterium seems to play a role in the development of ulceration of the non-glandular pars oesophagea [15]. In mice and Mongolian gerbil models of human gastric disease, experimental *H. suis* infection causes severe gastric pathology [4,16,17], including gastritis, parietal cell necrosis and the development of gastric MALT lymphoma-like lesions, resembling the lesions observed in *H. suis*-infected humans.

Previous studies have shown that this bacterium lacks a homologue for several virulence factors of *H. pylori*, such as the *cytotoxin associated genes* pathogenicity island (*cag*PAI) and the vacuolating cytotoxin (VacA) [18]. We were, however, capable of identifying the γ-glutamyl transpeptidase (GGT) as an important virulence factor of *H. suis*. This enzyme has been described to cause gastric epithelial cell damage [19] and modulation of lymphocyte proliferation [20] through the interaction of the enzyme with two of its substrates, L-glutamine and reduced glutathione, making it the first identified and investigated *H. suis* virulence determinant.

The role of GGT during *H. pylori* infection *in vivo* has been investigated in mice. Conflicting conclusions have been drawn regarding the importance of GGT for colonization. Some groups have concluded that *H. pylori* GGT is required for persistent infection in mice [21], while others have made contrary conclusions [22]. In addition, there is accumulating evidence that *Helicobacter* GGT is a crucial virulence factor involved in immune evasion and immune tolerance [23-25].

Currently, it is unknown if and how *H. suis* GGT influences the course of *H. suis* infection *in vivo*. The aim of the present study was to extend our previous *in vitro* findings with *H. suis* GGT, and to study the role of this virulence factor in the pathogenesis of *H. suis* infection *in vivo*. At the same time, we aimed at comparing its relative importance with that of the GGT of *H. pylori*. The current experiments were performed in BALB/c mice and outbred Mongolian gerbils, since these animal models have indeed been shown to be valuable tools to investigate the role of *Helicobacter* species in gastric pathology. Typically, in Mongolian gerbils, a more rapid and severe development of gastric lesions can be observed compared to mice [4,26,27].

## Material and methods

### Animal and bacterial strains

Sixty 4-week-old, female specific-pathogen-free (SPF) BALB/c mice were purchased from Harlan NL (Horst, The Netherlands). Twenty-five 4-week-old, female SPF outbred Mongolian gerbils (Crl:MON) were obtained from Charles River Laboratories (Lille, France).

For *H. suis* infection in mice and Mongolian gerbils, strain HS5cLP was used. This strain has been isolated in 2008 from the stomach of a slaughterhouse pig [28]. For experimental *H. pylori* infection in Mongolian gerbils, strain PMSS1 [29] was used, since this strain has no history of *in vivo* adaptation in mice, in contrast to the mouse-adapted strain SS1. In BALB/c mice, *H. pylori* strain SS1 [29] was used, since strain PMSS1 has previously been demonstrated not to be able to colonize the stomach of BALB/c mice [29].

### Construction of isogenic *ggt* mutant strains of *H. suis* and *H. pylori*

An isogenic *H. suis ggt* mutant strain (HS5cLPΔ*ggt*) was prepared as described previously [20]. The isogenic *ggt* mutant strain of *H. pylori* was obtained using the same strategy as for creation of the *H. suis* isogenic *ggt* mutant, except that a kanamycin resistance cassette was used instead of a chloramphenicol resistance cassette [20]. Very briefly, deletion of *ggt* in *H. pylori* SS1 and PMSS1 was introduced by allelic exchange using pBluescript II SK (+) phagemid vector (Agilent Technologies, California, USA) in which ~440 bp of the 5′ –end and ~430 bp of the 3′ –end of the target gene and the kanamycin resistance cassette from plasmid pKD4 [30] were ligated through a PCR-mediated strategy with 2 cycles of inverse PCR and fusion PCR [20]. All primers used for PCR-mediated construction of the recombinant plasmids are shown in Table 1*.* The resultant plasmid was amplified in XL1-Blue MRF′ *E. coli* (Agilent Technologies) and used as a suicide plasmid in *H. pylori* SS1 and PMSS1 (a kind gift from Sara Lindén and Anne Muller, respectively). The *H. pylori* SS1 *ggt* mutant (SS1Δ*ggt*) and *H. pylori* PMSS1 *ggt* mutant (PMSS1Δ*ggt*) were obtained by electrotransformation [31] or natural transformation [32] as described previously. Finally, bacteria were selected on columbia agar plates (Oxoid, Basingstoke, UK) with Vitox supplement (Oxoid), 5% (v/v) defibrinated sheep blood (E&O Laboratories Ltd, Bonnybridge, UK), and kanamycin (25 μg/mL). The plates were incubated for 5–9 days. The isogenic *ggt* mutants were verified by a GGT activity assay [19], PCR and nucleotide sequencing.

**Table 1:** **Primers used for construction of the** ***H. pylori ggt*** **isogenic mutant strains**

**Primer name**	**Sequence (5′- 3′)**	**Primer use**
pBlue linear Fwd 1	GGGGATCCACTAGTTCTAGAGCG	Linearization of plasmid
pBlue linear Rev1	CGGGCTGCAGGAATTCGATATCAAG	Linearization of plasmid
HpGGT-flank_fusion1F	CTTGATATCGAATTCCTGCAGCCCGTAACCGGTAAAATCAACACGGACGC	Amplification *H. pylori ggt* and partial up- and downstream flanking genes
HpGGT-flank_fusion1R	CGCTCTAGAACTAGTGGATCCCCGCGCTCTTATAAAAAGAAGCCGC	Amplification *H. pylori ggt* and partial up- and downstream flanking genes
pBluelinear_Hpggtflank1F	CCAAGGAAAGAATTTTAATCCTATTTAG	Linearization of the recombinant plasmid
pBluelinear_Hpggtflank1R	CTGTTTTCCTTTCAATCAACAATAATC	Linearization of the recombinant plasmid
Hpkana_fusion_1F	ATTATTGTTGATTGAAAGGAAAACAGATGATTGAACAAGATGGATTGC	Amplification kanamycin resistance gene
Hpkana_fusion_1R	CTAAATAGGATTAAAATTCTTTCCTTGGTCAGAAGAACTCGTCAAGAAG	Amplification kanamycin resistance gene
T7 prom3	TAATACGACTCACTATAGGG	Sequencing
M13R	CAGGAAACAGCTATGAC	Sequencing

### Culture conditions of bacterial strains

Wild-type (WT) *H. suis* strain HS5cLP was grown for 48 h as described previously [29]. HS5cLPΔ*ggt* bacteria were grown under the same conditions as strain HS5cLP, except that the cultivation plates were supplemented with chloramphenicol (30 μg/mL) as described previously [20].

WT *H. pylori* strains SS1 and PMSS1 were grown on Columbia agar plates containing 5% (v/v) defibrinated sheep blood for 48–72 h at 37 °C under microaerobic conditions as described previously [29]. Subsequently, colonies were picked up and cultured in Brucella broth supplemented with Vitox (Oxoid) and 5% fetal calf serum (HyClone) on a rotational shaker under microaerobic conditions (16 h, 125 rpm). SS1Δ*ggt* and PMSS1Δ*ggt* strains were cultured under the same conditions as the corresponding WT strains on plates supplemented with kanamycin (25 μg/mL).

### Experimental design

Upon arrival, sixty BALB/C mice and twenty-five Mongolian gerbils were divided into 5 groups, and the animals were allowed to acclimate to the new environment for 1 week. Animals were inoculated intragastrically 3 times at 48 h intervals. Animals from group 1 and 2 (both mice and Mongolian gerbils) were inoculated with Brucella broth containing 8 × 10^7^ viable bacteria of strains HS5cLP and HS5cLPΔ*ggt*, respectively. Animals in group 3 and 4 were inoculated with Brucella broth containing 3 × 10^8^ viable bacteria of strains SS1 and SS1Δ*ggt* (mice) or 1 × 10^9^ viable bacteria of strains PMSS1 and PMSS1Δ*ggt* (gerbils). Animals in the fifth group were inoculated with Brucella broth and served as uninfected controls. For mice, at 4 weeks, 9 weeks and 6 months post infection (pi), 4 animals from each group were euthanized by cervical dislocation under isoflurane anaesthesia. For Mongolian gerbils, all animals were sacrificed at 9 weeks pi. The stomachs of the animals were resected for further processing as described previously [27,29].

Animal experiments were approved by the Ethical Committee of the Faculty of Veterinary Medicine, Ghent University, Belgium (EC2013/29).

### Histopathological examination and immunohistochemistry (IHC)

Three longitudinal strips of gastric tissue from mice and Mongolian gerbils were cut from the oesophagus to the duodenum along the greater curvature. Tissue was fixed in 4% phosphate buffered formaldehyde, processed by standard methods and embedded in paraffin for light microscopy. Five serial sections of 5 μm were cut. The first section was stained with haematoxylin/eosin (H&E) to score the degree of gastritis according to the Updated Sydney System with some modifications [33]. After deparaffinization and rehydration for the remaining sections, heat-induced antigen retrieval was performed in citrate buffer (pH = 6.0). In order to block endogenous peroxidase activity and non-specific reactions, all the slides were incubated with 3% H_2_O_2_ in methanol (5 min) and 30% goat serum (30 min), respectively. For the differentiation between T and B lymphocytes, CD3 and CD20 antigens were stained on sections two and three, using a polyclonal rabbit anti-CD3 antibody (1/100; DakoCytomation, Glostrup, Denmark) and a polyclonal rabbit anti-CD20 antibody (1/25; Thermo Scientific, Fremont, USA), respectively. These sections were further processed with Envision + System-HPR (DAB) (DakoCytomation) for use with rabbit primary antibodies. On the fourth and fifth section, epithelial cell proliferation and the number of parietal cells were determined by IHC staining, using a mouse monoclonal anti-Ki67 antibody (1/25; Menarini Diagnostics, Zaventem, Belgium) and mouse monoclonal anti-hydrogen potassium ATPase β-subunit (H^+^/K^+^ ATPase) antibody (1/25 000; Abcam Ltd, Cambridge, UK), respectively. Subsequent visualization was done with Envision + System-HPR (DAB) (DakoCytomation) for use with mouse primary antibodies. Quantification of T cells, B cells and epithelial cells were performed as described previously [4]. Briefly, the numbers of cells belonging to defined cell populations (T cells, B cells, and epithelial cells) were determined by counting the positive cells in five randomly chosen High Power Fields (magnification: × 400), both in the antrum and corpus region.

In order to assess the possible development of pseudopyloric metaplasia induced by *Helicobacter* infection, alcian blue-periodic acid-schiff stain staining (AB/PAS) was performed.

### Quantification of colonizing bacteria in the stomach of mice and Mongolian gerbils

Strips of gastric tissue containing all regions for mice and separate pieces (antrum and corpus) for Mongolian gerbils were stored in 0.5 mL RNA*later* solution (Ambion, Austin, TE, USA) at −70 °C until RNA and DNA extraction. Quantitative Real-Time PCR (qRT-PCR) was used to determine the number of colonizing bacteria in the gastric tissue as described previously [29,34].

### RNA extraction and reverse transcription

qRT-PCR was used to determine gene expression in the gastric tissue from mice and Mongolian gerbils. Total RNA was extracted using the RNeasy Mini Kit (Qiagen, Hilden, Germany) according to the manufacturer’s instructions. The concentration of RNA was measured using a NanoDrop spectrophotometer (Isogen Life Science, PW De Meern, Utrecht, The Netherlands). The purity of the RNA was evaluated with the Experion automated electrophoresis system using StdSens RNA chips (Bio-Rad, Hercules CA, USA). The RNA concentration from all samples was adjusted to 1 μg/μL and cDNA was synthesized immediately after RNA purification using the iScript™ cDNA Synthesis Kit (Bio-Rad).

### Design and validation of primers and determination of gene expression

The housekeeping genes *H2afz, PPIA* and *HPRT* were included as reference genes for mice [29]. For Mongolian gerbils, a set of reference genes was tested based on the fact that they are extensively used in other animal species. Primers were designed based on the conserved regions of *ACTB, β-actin, RPS18*, *GAPDH*, *HPRT1*, *SDHA* and *UBC* complete or partial coding sequences available for humans, pigs, mice and rats.

The mRNA expression levels of various cytokines (IFN-γ, IL-4, IL-5, IL-17, IL-1β, IL-6, IL-10), previously shown to be differentially expressed during *H. suis* infection, as well as other genes (Foxp3, CXCL13, ASCT2, ATP4a, and ATP4b) were quantified using SYBR Green based RT-PCR with iQ™ SYBR Green Supermix. Reactions were performed using a CFX96 RT PCR System in a C1000 Thermal Cycler (Bio-Rad) as described previously [29]. All reactions were performed in 12 μL volumes containing 0.05 μL of each primer (1.25 pmol/μL), 6 μL iQ™ SYBR Green Supermix, 3.9 μL HPLC water and 2 μL cDNA. The experimental program consisted of 95 °C for 15 min, followed by 40 cycles of denaturation at 95 °C for 20 s, annealing at 60 °C for 30 s, and extension at 72 °C for 30 s. The threshold cycle values (Ct) were normalized to the geometric means of the reference genes and the normalized mRNA levels of all target genes were calculated using the method of 2^−ΔΔCt^ [35].

Due to the unavailability of gene information for Forkhead/winged helix transcription factor (Foxp3) and the chemokine CXC ligand 13 (CXCL13) from Mongolian gerbils, primers were designed based on the conserved regions of Foxp3 and CXCL13 complete or partial coding sequences available for humans, pigs, mice and rats with the same strategy as described above. The mRNA expression levels of Foxp3 and CXCL13 were determined using the same method as described above. Sequence information of all the primers for mice and for Mongolian gerbils is shown in Tables 2 and 3.

**Table 2:** **List of genes and primers used for qRT-PCR in Mongolian gerbils**

**Gene**	**Primer**	**Sequence (5′- 3′)**	**References**
*Foxp3*	sense	GCCCCTMGTCATGGTGGCA	This study
	antisense	CCGGGCCTTGAGGGAGAAGA	
*CXCL13*	sense	GAATGGCTGCCCCAAAACTGAA	This study
	antisense	TCACTGGAGCTTGGGGAGTTGAA	
*GAPDH*	sense	AACGGGAAGCTCACTGGCATG	This study
	antisense	CTGCTTCACCACCTTCTTGATGTCA	
*HPRT1*	sense	GCCCCAAAATGGTTAAGGTTGCA	This study
	antisense	TCAAGGGCATATCCAACAACAAAC	
*RPS18*	sense	CGAGTACTCAACACCAACATCGATGG	This study
	antisense	ATGTCTGCTTTCCTCAACACCACATG	
*IL-1β*	sense	GGCAGGTGGTATCGCTCATC	[64]
	antisense	CACCTTGGATTTGACTTCTA	
*IFN-γ*	sense	CCATGAACGCTACACACTGCATC	[65]
	antisense	GAAGTAGAAAGAGACAATCTGG	
*IL-5*	sense	AGAGAAGTGTGGCGAGGAGAGACG	[27]
	antisense	ACAGGGCAATCCCTTCATCGG	
*IL-6*	sense	GAGGTGAAGGATCCAGGTCA	[66]
	antisense	GAGGAATGTCCTCAGCTTGG	
*IL-10*	sense	GGTTGCCAAGCCTTATCAGA	[27]
	antisense	GCTGCATTCTGAGGGTCTTC	
*IL-17*	sense	AGCTCCAGAGGCCCTCGGAC	[64]
	antisense	AGGACCAGGATCTCTTGCTG	
*ATP4b*	sense	GGGGGTAACCTTGAGACCTGATG	[27]
	antisense	AAGAAGTACCTTTCCGACGTGCAG	
*β-actin*	sense	TCCTCCCTGGAGAAGAGCTA	[66]
	antisense	CCAGACAGCACTGTGTTGGC

**Table 3:** **List of genes and primers used for qRT-PCR in mice**

**Gene**	**Primer**	**Sequence (5′- 3′)**	**References**
*IL-1β*	sense	GGGCCTCAA AGGAAAGAATC	[29]
	antisense	TACCAGTTGGGGAACTCTGC	
*IFN-γ*	sense	GCGTCATTGAATCACACCTG	[29]
	antisense	TGAGCTCATTGAATGCTTGG	
*IL-4*	sense	ACTCTTTCGGGCTTTTCGAT	[29]
	antisense	AAAAATTCATAAGTTAAAGCATGGTG	
*IL-10*	sense	ATCGATTTCTCCCCTGTGAA	[29]
	antisense	CACACTGCAGGTGTTTTAGCTT	
*IL-17*	sense	TTTAACTCCCTTGGCGCAAAA	[29]
	antisense	CTTTCCCTCCGCATTGACAC	
*Foxp3*	sense	GCCCCTMGTCATGGTGGCA	This study
	antisense	CCGGGCCTTGAGGGAGAAGA	
*CXCL13*	sense	CTCTCCAGGCCACGGTATT	[67]
	antisense	TAACCATTTGGCACGAGGAT	
*ATP4a*	sense	TGCTGCTATCTGCCTCATTG	[68]
	antisense	GTGCTCTTGAACTCCTGGTAG	
*ATP4b*	sense	AACAGAATTGTCAAGTTCCTC	[68]
	antisense	AGACTGAAGGTGCCATTG	
*HPRT*	sense	CAGGCCAGACTTTGTTGGAT	[29]
	antisense	TTGCGCTCATCTTAGGCTTT	
*PPIA*	sense	AGCATACAGGTCCTGGCATC	[29]
	antisense	TTCACCTTCCCAAAGACCAC	
*H2afz*	sense	CGTATCACCCCTCGTCACTT	[29]
	antisense	TCAGCGATTTGTGGATGTGT	

### Statistical analysis

Differences in colonization capacity were analyzed using a non-parametric Mann–Whitney *U* test. Differences in lymphocytic infiltration, cytokine expression and IHC analysis were assessed with one-way ANOVA followed by a Bonferroni post hoc test. Statistical analyses were performed using SPSS Statistics 20 software (IBM). Pair-wise comparisons were done for each individual time-point and on pooled data using time as stratification factor. *P* values less than 0.05 were considered statistically significant. All data are expressed as mean ± SD. All the figures were created using GraphPad Prism5 software (GraphPad Software Inc., San Diego, CA, USA).

## Results

### Colonization density

**Figure 1:**
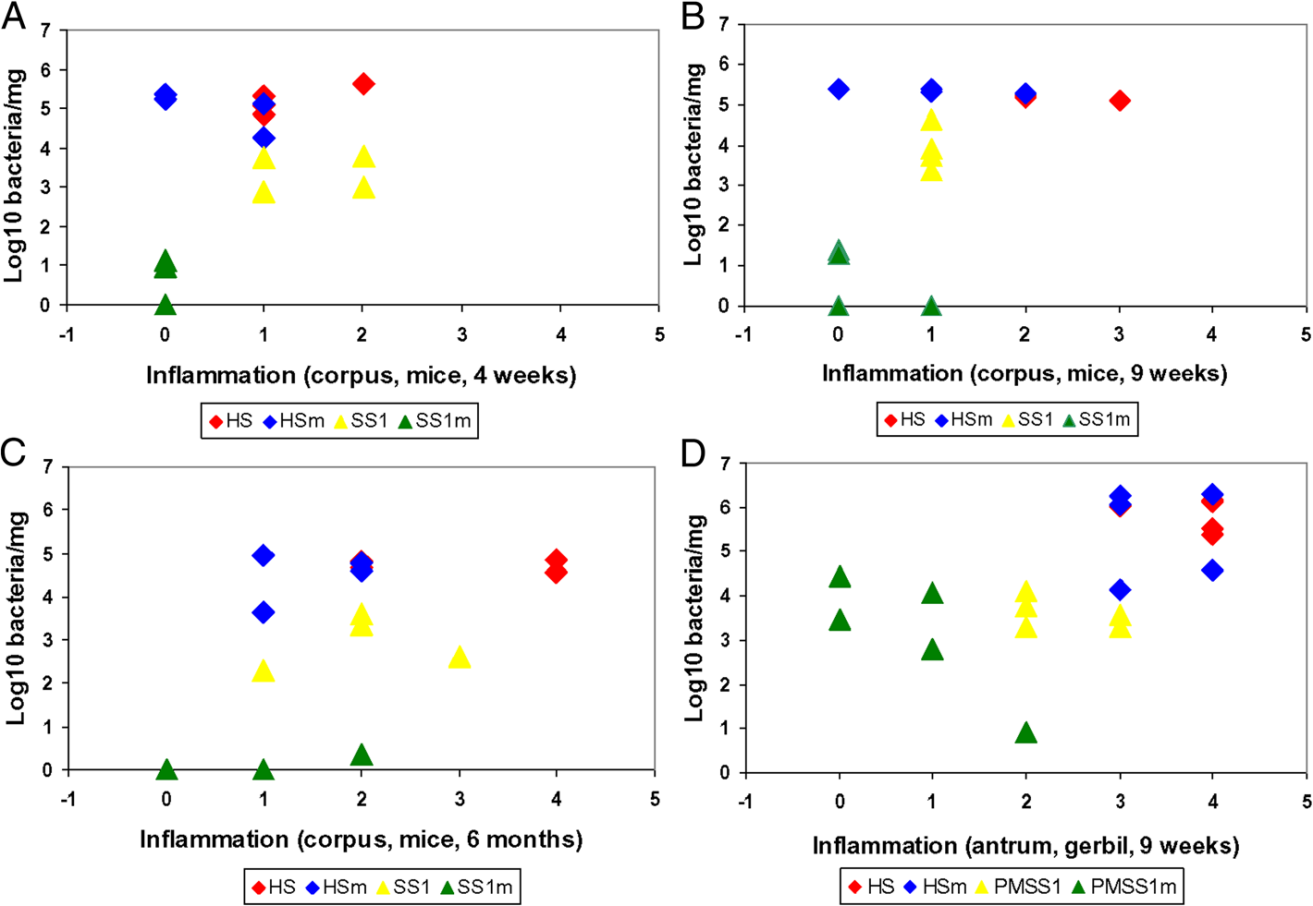
**Correlation between bacterial colonization capacity and inflammation score in the stomach of mice and Mongolian gerbils.** The colonization capacity is shown as log10 values of *H. suis* or *H. pylori* per mg tissue, determined with qRT-PCR in the corpus of mice **(A-C)** and antrum of Mongolian gerbils **(D)**. 0, no infiltration with mononuclear and/or polymorphonuclear cells; 1, very mild diffuse infiltration with mononuclear and/or polymorphonuclear cells or the presence of one small (20–50 cells) aggregate of inflammatory cells; 2, mild diffuse infiltration with mononuclear and/or polymorphonuclear cells or the presence of one small (50–200 cells) aggregate of inflammatory cells; 3, moderate diffuse infiltration with mononuclear and/or polymorphonuclear cells and/or the presence of 2–4 inflammatory aggregates; 4, marked diffuse infiltration with mononuclear and/or polymorphonuclear cells and/or the presence of at least five inflammatory aggregates. HS vs. HSm: Colonization: *p* > 0.05; Inflammation: *p* < 0.05. SS1 vs. SS1m: Colonization: *p* < 0.05; Inflammation: *p* < 0.05. PMSS1 vs. PMSS1m: Colonization: *p* > 0.05; Inflammation: *p* < 0.05. HS: animals infected with WT *H. suis* srain HS5cLP; HSm: animals infected with *H. suis* strain HS5cLPΔ*ggt*; SS1: animals infected with WT *H. pylori* SS1; SS1m: animals infected with *H. pylori* SS1Δ*ggt*; PMSS1: animals infected with WT *H. pylori* PMSS1; PMSS1m: animals infected with *H. pylori* PMSS1Δ*ggt*.

All control animals were negative for *Helicobacter*. Results of infected animals showed that WT *H. suis* can persistently colonize the mouse stomach with colonization levels as high as 5.42 × 10^4^ (±1.46 × 10^4^) bacteria/mg gastric tissue even at 6 months pi (Figure 1C). *H. pylori* strain SS1 was shown to colonize the mouse stomach at a much lower bacterial density, being 1.68 × 10^3^ (±1.73 × 10^3^) bacteria/mg tissue at 6 months pi (Figure 1C, *p* < 0.05).

Interestingly, *H. suis* strain HS5cLPΔ*ggt* was able to colonize the corpus of the stomach of the mice to a similar extent as the WT strain, and this was observed for all timepoints (Figures 1A-1C). In contrast, *H. pylori* strain SS1Δ*ggt* was shown to have an impaired colonization capacity in mice at all three timepoints (Figures 1A-1C, *p* < 0.05). Similar colonization data were demonstrated in the antrum of *Helicobacter* infected-mice at all three timepoints (data not shown).

Both the HS5cLP and HS5cLPΔ*ggt* strain successfully colonized the antrum and corpus of the stomach of Mongolian gerbils, although colonization rates were much lower in the corpus compared to the antrum. No statistically significant differences were observed between both strains (Figure 1D, *p* > 0.05). *H. pylori* strain PMSS1Δ*ggt* was able to colonize the antrum and corpus of the stomach at similar levels compared to PMSS1 (Figure 1D, *p* > 0.05), although 2 out 5 Mongolian gerbils were negative for the presence of PMSS1Δ*ggt* in the corpus of the stomach (data not shown).

### Infection-induced inflammation

All control mice and gerbils showed normal gastric histomorphology at all timepoints. The correlation between inflammation scores and bacterial colonization is displayed in Figure 1.

**Figure 2:**
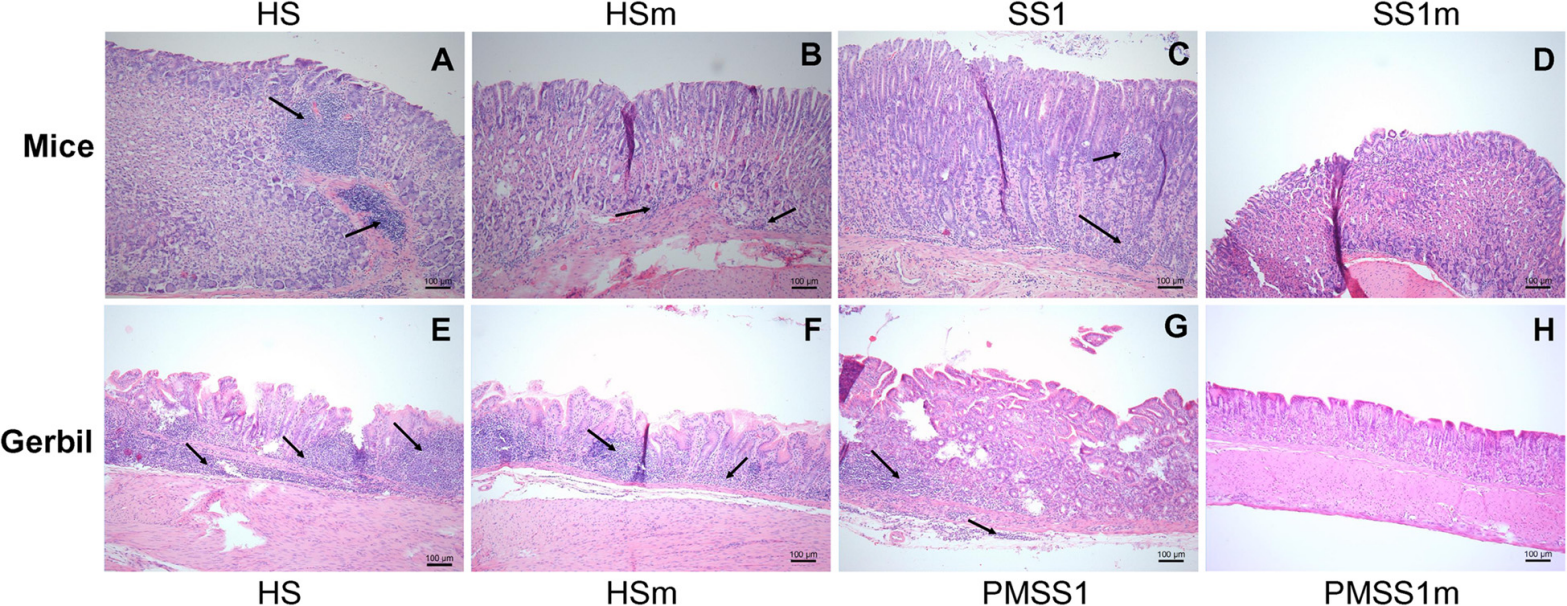
**H&E staining of stomach sections from** ***Helicobacter*** **-infected mice and Mongolian gerbils.** Representative micrographs of H&E stained sections shown here were taken from mice orally inoculated with *H. suis* HS5cLP **(A)**, *H. suis* HS5cLPΔ*ggt* **(B)**, *H. pylori* SS1 **(C)** and *H. pylori* SS1Δ*ggt* **(D)** at 6 months post inoculation and Mongolian gerbils orally challenged with *H. suis* HS5cLP **(E)**, *H. suis* HS5cLPΔ*ggt* **(F)**, *H. pylori* PMSS1 **(G)** and *H. pylori* PMSS1Δ*ggt* **(H)** at 9 weeks post inoculation. Arrows indicate the presence of inflammatory cells, inflammatory aggregates, lymphocytic infiltration, or lymphocytic follicles. HS: animals infected with WT *H. suis* strain HS5cLP; HSm: animals infected with *H. suis* strain HS5cLPΔ*ggt*; SS1: animals infected with WT *H. pylori* SS1; SS1m: animals infected with *H. pylori* SS1Δ*ggt*; PMSS1: animals infected with WT *H. pylori* PMSS1; PMSS1m: animals infected with *H. pylori* PMSS1Δ*ggt*; WT: wild-type. Original magnification: 100 × .

Compared to mice with WT strain infection, infection with *H. suis* strain HS5cLPΔ*ggt* generally induced significantly less overall inflammation both in the antrum (*p* < 0.01) and corpus (*p* < 0.01), whereas only in the corpus region (*p* < 0.01), infection with *H. pylori* strain SS1Δ*ggt* induced less inflammation, compared to that seen in WT strain infected mice. At 6 months pi, the corpus region in 2 out of 4 mice with HS5cLP infection contained large lymphoid aggregates or lymphoid follicles accompanied by destruction of the normal mucosal architecture (Figure 2A), which was not observed in animals from other groups.

For Mongolian gerbils, infection with HS5cLP or PMSS1 induced severe antrum-dominant gastritis with formation of lymphocytic aggregates in the lamina propria and/or sub-mucosa of the stomach (Figures 1D, 2E, and 2G). No significant differences were observed between the WT and mutant strain of *H. suis* with respect to the inflammatory response induced in gerbils (Figures 1D, 2E and 2 F), although all animals infected with strain HS5cLP showed inflammation in the corpus region, whereas this was only the case for some animals infected with HS5cLPΔ*ggt* (data not shown). In one gerbil infected with *H. suis* strain HS5cLP, a pronounced inflammatory response was observed, in which more than 65% of the area in the lamina propria and submucosa of the antrum was densely infiltrated with inflammatory cells, fused lymphoid aggregates and lymphoid follicles (Additional file 1).

Inflammation induced by *H. pylori* strain PMSS1Δ*ggt* in the antrum of gerbils was less severe compared to that seen in WT infected animals (*p* < 0.05) (Figures 1D, 2G and 2H).

### Inflammatory cell infiltration

**Figure 3:**
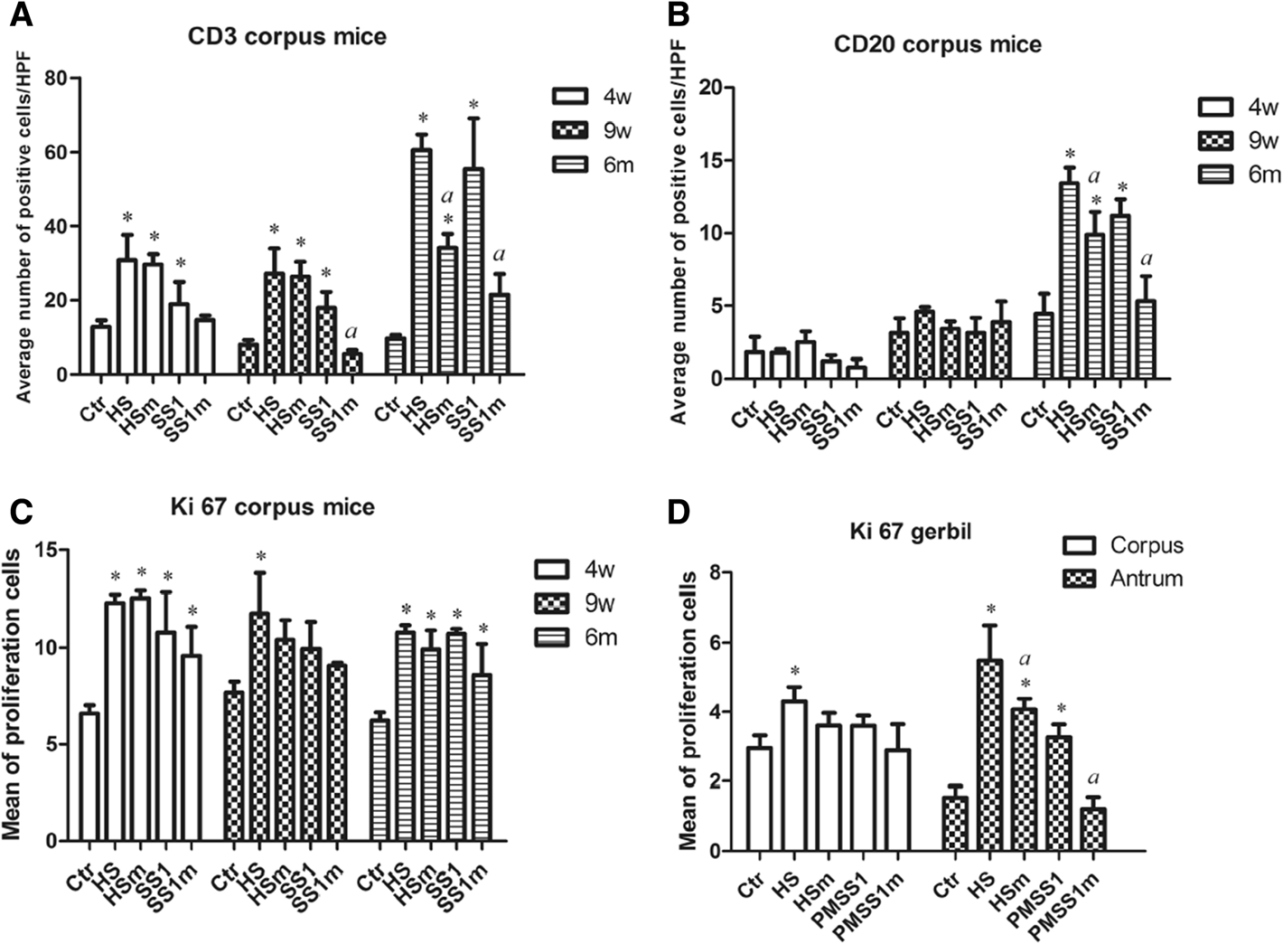
**Quantitative analysis of defined cell populations with immunohistochemistry. (A-B)** Shown are the average (± SD) numbers of cells/ High Power Field, including T cells (CD3-positive) and B cells (CD20-positive) in the corpus of the stomach of mice. **(C-D)** Shown are the average (± SD) numbers of epithelial cells in five randomly chosen microscopic fields at the level of the gastric pits in the stomach from mice and Mongolian gerbils. An * represents a statistically significant difference (*p* < 0.05) between infected and control groups. An *a* represents a statistically significant difference (*p* < 0.05) between WT *Helicobacter* infected groups and isogenic *ggt* mutant infected groups. Ctr: animals from control group; HS: animals infected with WT *H. suis* strain HS5cLP; HSm: animals infected with *H. suis* strain HS5cLPΔ*ggt*; SS1: animals infected with WT *H. pylori* SS1; SS1m: animals infected with *H. pylori* SS1Δ*ggt*; PMSS1: animals infected with WT *H. pylori* PMSS1; PMSS1m: animals infected with *H. pylori* PMSS1Δ*ggt*; WT: wild-type; 3w: 3 weeks post infection; 9w: 9 weeks post infection; 6 m: 6 months post infection.

In general, an increase of T cell numbers was observed in the corpus (Figure 3A, *p* < 0.05) of mice infected with *H. suis* strain HS5cLP and *H. pylori* strain SS1 at all three timepoints. Compared to the mice infected with WT *H. suis*, HS5cLPΔ*ggt* induced a lower T cell response in the corpus at 6 months pi (*p* < 0.01). *H. pylori* strain SS1Δ*ggt* induced a reduced T cell response in the corpus region (*p* < 0.01) compared to WT infected animals, at both 9 weeks and 6 months pi (Figure 3A). Similar results were observed in the antrum of mice (data not shown).

An increase of B cell numbers was observed in the corpus mucosa of mice infected with strain HS5cLP (*p* < 0.01) and SS1 (*p* < 0.01) at 6 months pi (Figure 3B). Compared to the WT *H. suis* infected mice, HS5cLPΔ*ggt* induced a lower B cell response in the corpus region of mice at 6 months pi (*p* < 0.05) and a similar reduction was observed in SS1Δ*ggt* infected mice (*p* < 0.01) (Figure 3B).

**Figure 4:**
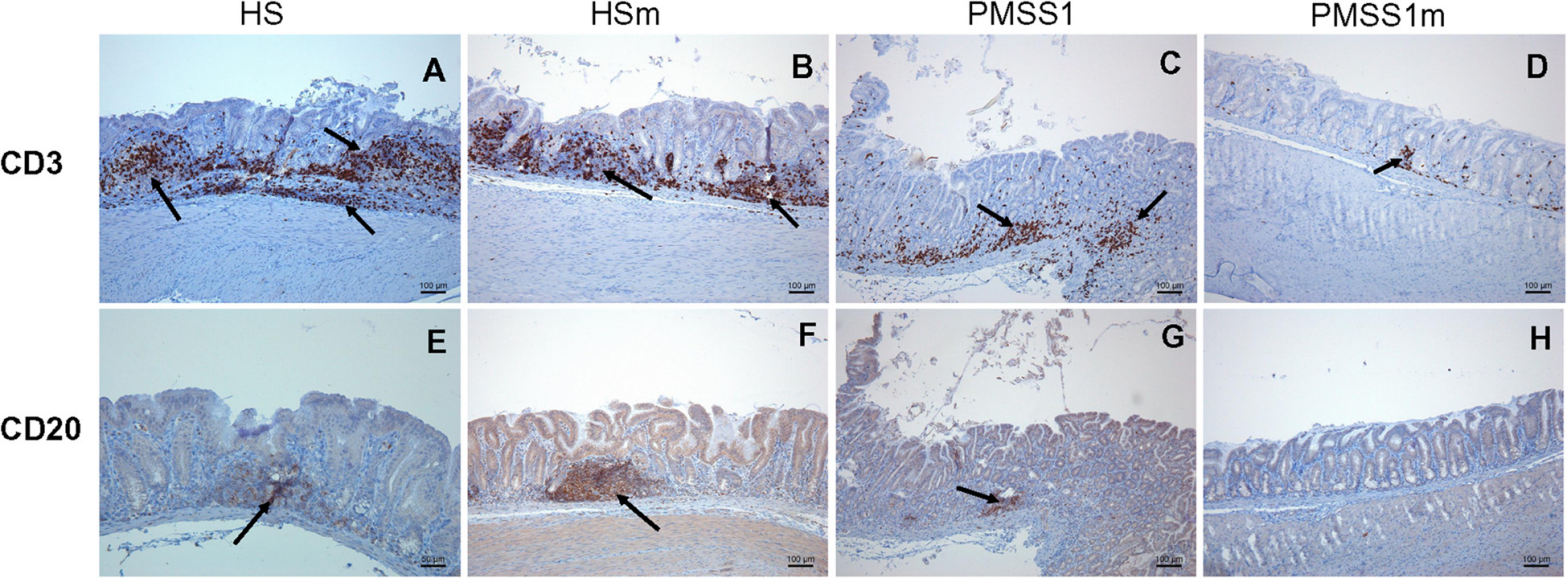
**Gastric inflammation of** ***Helicobacter*** **-infected Mongolian gerbils.** CD3 staining of the antrum of the stomach from Mongolian gerbils inoculated with *H. suis* HS5cLP **(A)**, *H. suis* HS5cLPΔ*ggt* **(B)**, *H. pylori* PMSS1 **(C)** and *H. pylori* PMSS1Δ*ggt* **(D)** at 9 weeks post inoculation, showing T-lymphocytes (brown). CD20 staining of the antrum of a gerbil infected with WT *H. suis* HS5cLP **(E)**, *H. suis* HS5cLPΔ*ggt* **(F)**, WT *H. pylori* PMSS1 **(G)** and *H. pylori* PMSS1Δ*ggt* **(H)** at 9 weeks post inoculation, showing B lymphocytes (brown) in germinal centers of lymphoid follicles (arrows) or lymphoid aggregates (arrows). HS: animals infected with WT *H. suis* strain HS5cLP; HSm: animals infected with *H. suis* strain HS5cLPΔ*ggt*; PMSS1: animals infected with WT *H. pylori* PMSS1; PMSS1m: animals infected with *H. pylori* PMSS1Δ*ggt*; WT: wild-type. Original magnification: 100 × .

For Mongolian gerbils, an exact quantification of T and B lymphocytes was not performed since the inflammation was characterized by a marked diffuse infiltration with large numbers of lymphocytes and large inflammatory aggregates. Histopathological analysis showed a pronounced increase of T cell numbers as well as lymphocytic aggregates and follicles in the lamina propria and tunica submucosa in all groups (Figures 4A-4D), although this was most pronounced in the antrum of both WT and mutant *H. suis* infected animals (Figures 4A-4B). T cell infiltration levels induced by PMSS1Δ*ggt* infection were lower compared to that seen in WT *H. pylori* infected animals (Figures 4C and 4D).

WT and mutant strains of *H. suis* induced similar levels of B cell infiltration, mainly in the centre of lymphocytic aggregates/follicles in the antrum (Figures 4E and 4 F). WT *H. pylori* induced mild B cell infiltration in the antrum of gerbils, whereas animals with PMSS1Δ*ggt* infection did not show an obvious B cell infiltration (Figures 4G and 4H). A marked proliferation of B cells in germinal centers was observed in gerbils infected with *H. suis* strains HS5cLP (Additional file 2A) and HS5cLPΔ*ggt* (Additional file 2B) but not in *H. pylori* infected animals.

### Epithelial cell-related changes

For mice, IHC staining did not reveal a clear decrease of the number of parietal cells in the stomach, except for mice infected with *H. suis* strain HS5cLP for 6 months (*p* < 0.05). For Mongolian gerbils, a clear loss of parietal cells was only observed in the transition zone between corpus and antrum in *H. suis* strain HS5cLP (Additional file 3B) and HS5cLPΔ*ggt* (Additional file 3C) infected animals, but not in *H. pylori* PMSS1 or PMSS1Δ*ggt* infected animals (Additional files 3D and 3E).

Data on gastric epithelial cell proliferation in the corpus region are summarized in Figures 3C and 3D. Compared to control mice, an increased epithelial cell proliferation was seen in the corpus (Figure 3C, *p* < 0.05) of HS5cLP infected mice at all timepoints, and a similar increase was observed for SS1 infected mice (Figure 3C, *p* < 0.05). In general, mice infected with *H. suis* and *H. pylori* strains mutated for the GGT revealed somewhat lower epithelial cell proliferation rates compared to WT strain infected mice (Figure 3C), which was, however, not statistically significant. Compared to WT strain infected Mongolian gerbils, both HS5cLPΔ*ggt* (*p* < 0.05) and PMSS1Δ*ggt* (*p* < 0.01) infected animals revealed a significantly lower level of epithelial cell proliferation in the antrum (Figure 3D).

AB/PAS staining showed that *H. suis* infection triggered the development of pseudopyloric metaplasia to a varying degree in the corpus region of mice at 6 months pi (Additional files 4B and 4C). Compared to WT *H. suis* infection, infection with HS5cLPΔ*ggt* in general led to less obvious regions affected by pseudopyloric metaplasia. Infection with WT *H. pylori* also induced pseudopyloric metaplasia to a varying degree in the corpus region of mice at 6 months pi (Additional file 4D), whereas strain SS1Δ*ggt* did not (Additional file 4E).

### Cytokine secretion in response to bacterial infection

**Figure 5:**
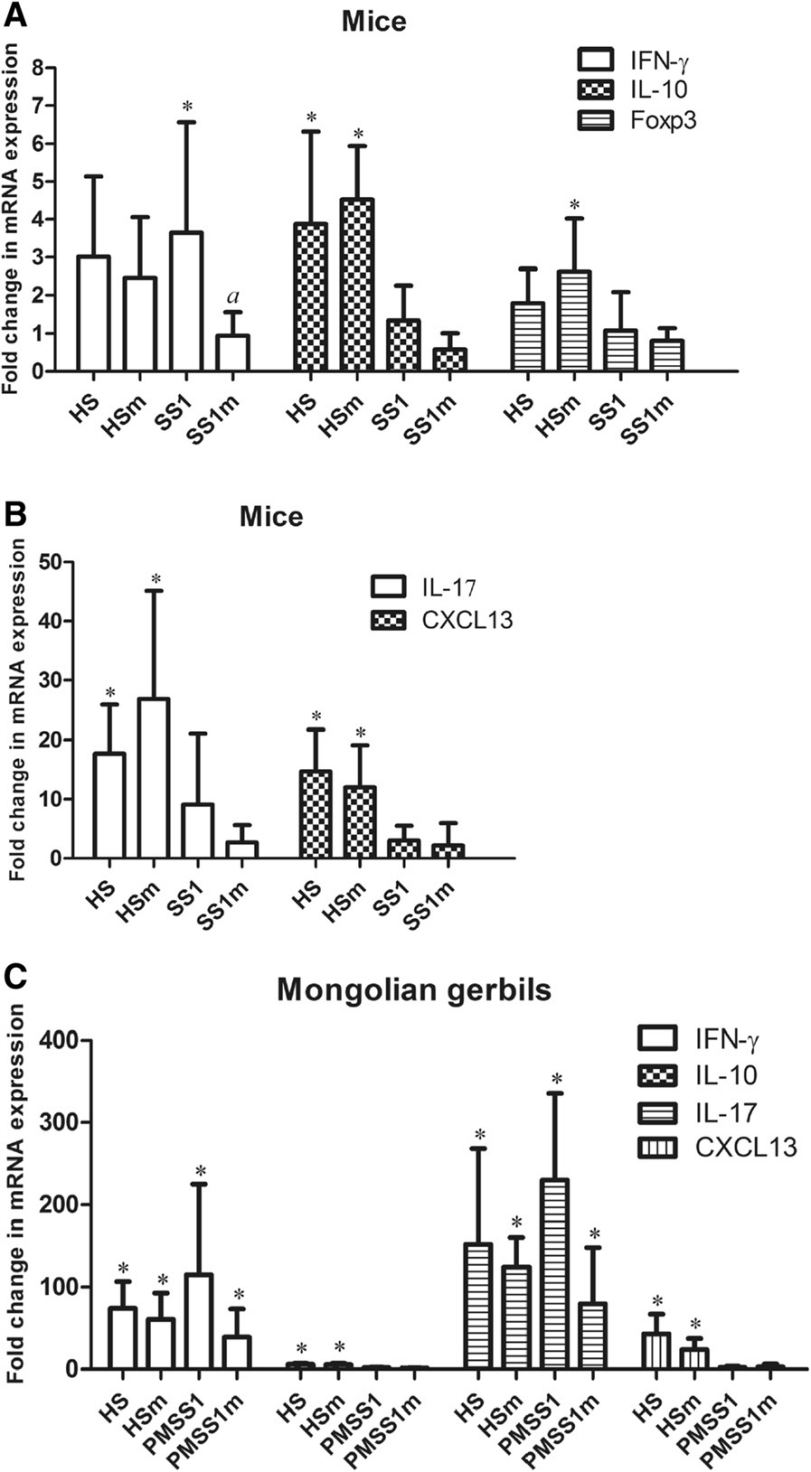
**Cytokine expression patterns in the stomach of mice and Mongolian gerbils infected with** ***H. suis*** **and** ***H. pylori*** **.** Shown are the mean fold changes of mRNA expression in infected mice **(A-B)** and gerbils **(C)** for IFN-γ, IL-10, Foxp3, IL-17, CXCL13. The mean fold changes in the relevant uninfected control groups is equal to 1. An * indicates a statistically significant difference (*p* < 0.05) between infected and control groups. An *a* indicates a statistically significant difference (*p* < 0.05) between WT *Helicobacter* infected groups and isogenic *ggt* mutant infected groups. HS: animals infected with WT *H. suis* strain HS5cLP; HSm: animals infected with *H. suis* strain HS5cLPΔ*ggt*; SS1: animals infected with WT *H. pylori* SS1; SS1m: animals infected with *H. pylori* SS1Δ*ggt*; PMSS1: animals infected with *H. pylori* PMSS1; PMSS1m: animals infected with *H. pylori* PMSS1Δ*ggt*; WT: wild-type.

**Figure 6:**
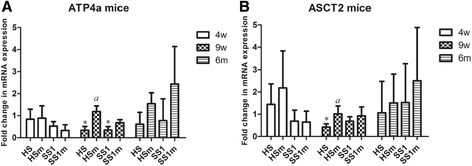
**Expression of epithelial cell-associated factors.** Shown are the mean fold changes of mRNA expression in infected mice for ATP4a **(A)** and ASCT2 **(B)**. The mean fold changes in the relevant uninfected control groups is equal to 1. An * indicates a statistically significant difference (*p* < 0.05) between infected and control groups. An *a* indicates a statistically significant difference (*p* < 0.05) between WT *Helicobacter* infected groups and isogenic *ggt* mutant infected groups. HS: animals infected with WT *H. suis* strain HS5cLP; HSm: animals infected with *H. suis* strain HS5cLPΔ*ggt*; SS1: animals infected with WT *H. pylori* SS1; SS1m: animals infected with *H. pylori* SS1Δ*ggt*; PMSS1: animals infected with *H. pylori* PMSS1; PMSS1m: animals infected with *H. pylori* PMSS1Δ*ggt*; WT: wild-type; 3w: 3 weeks post infection; 9w: 9 weeks post infection; 6 m: 6 months post infection.

Data on gene expression levels are presented in Figures 5 and 6.

Primers for housekeeping genes of Mongolian gerbils were chosen based on the specificity and amplification efficiency of the primers, and stable expression levels of the genes. *β-actin*, *RPS18*, *GAPDH* and *HPRT1* were included as the final reference genes for qRT-PCR performed in gerbils.

### IFN-γ and IL-1β

In general, only *H. pylori* strain SS1 infection induced a significant up-regulation of the Th1 signature cytokine IFN-γ in mice (Figure 5A, *p* < 0.05). WT and mutant strains of *H. suis* (*p* < 0.01) and *H. pylori* (*p* < 0.01) induced a pronounced upregulation of IFN-γ expression in the antrum of infected Mongolian gerbils (Figure 5C), and no significant differences were observed between WT infected- and mutant infected animals.

No significant differences of IL-1β expression were observed between groups (data not shown). In Mongolian gerbils, similarly increased expression levels of IL-1β were seen in animals infected with WT and mutant strains of *H. suis* (data not shown).

### IL-4, IL-5, IL-6, IL-10

In general, compared to control mice, the expression of anti-inflammatory IL-10 was upregulated in *H. suis* strain HS5cLP and HS5cLPΔ*ggt* infected mice (Figure 5A, *p* < 0.01). A very similar expression pattern was observed for Foxp3 (Figure 5B, *p* < 0.01), an important cell marker of CD4^+^/CD25^+^ regulatory T cells (Tregs), which are one of the most important cell types secreting IL-10 [36].

In Mongolian gerbils, a clear increase of IL-10 expression, compared to control animals, was demonstrated both in the antrum of gerbils infected with strain HS5cLP (*p* < 0.01) and strain HS5cLPΔ*ggt* (Figure 5C, *p* < 0.01). Compared to control animals, no significant changes of IL-10 and Foxp3 expression levels were observed in animals infected with *H. pylori* (Figures 5A and 5C).

Compared to control animals, an upregulation of IL-6 expression was only demonstrated in gerbils with HS5cLP and HS5cLPΔ*ggt* infection, but no difference was observed between both groups (data not shown). No significant differences in expression between control animals and infected animals could be demonstrated for IL-4 and IL-5 (data not shown).

### IL-17

IL-17 is a Th17 response signature cytokine. A notable increase of IL-17 expression was generally observed in mice infected with WT *H. suis* (Figure 5B, *p* < 0.05). Similar expression levels were observed for HS5cLPΔ*ggt* infected mice (Figure 5B, *p* < 0.05).

In Mongolian gerbils, both WT and mutant *H. suis* and *H. pylori* infection generally induced increased levels of IL-17 expression (Figure 5C, *p* < 0.01). These levels were lower in HS5cLPΔ*ggt* and PMSS1Δ*ggt* infected gerbils compared to WT infected animals, which was, however, not statistically significant (Figure 5C, *p* > 0.05), most likely due to the limited number of animals in each group.

### CXCL13

CXCL13 plays an important role during the B-cell homing to follicles in lymph nodes and spleen and formation of gastric lymphoid follicles [37], and it is involved in the pathogenesis of *Helicobacter* infection [14,37]. In general, infection with both HS5cLP and HS5cLPΔ*ggt* induced a marked upregulation of CXCL13 in mice (Figure 5B, *p* < 0.01). Moreover, an even higher increase of CXCL13 expression levels was observed in the antrum of gerbils infected with *H. suis* strains HS5cLP and HS5cLPΔ*ggt* compared to control gerbils (Figure 5C, *p* < 0.01). No statistically significant differences of CXCL13 expression levels were observed between HS5cLP and HS5cLPΔ*ggt* infected animals (Figures 5B and 5C).

### Changes of epithelial cell-related factors in the stomach

The H^+^/K^+^ ATPase is responsible for gastric acid secretion by parietal cells [38]. Compared to uninfected control mice, a clear decrease of *Atp4a* (Figure 6A, *p* < 0.05) and *Atp4b* (*p* < 0.05, data not shown) mRNA expression levels was detected in the stomach of HS5cLP and SS1 infected mice at 9 weeks pi. In addition, a statistically higher expression of *Atp4a* (Figure 6A, *p* < 0.05) and *Atp4b* (*p* < 0.05, data not shown) was observed in HS5cLPΔ*ggt* infected mice compared to WT infected animals.

ASCT2 is an important glutamine transporter for the growth of epithelial cells and other cell types [39]. Compared to control animals, infection with *H. suis* strain HS5cLP resulted in a downregulation of ASCT2 expression in mice at 9 weeks pi (Figure 6B, *p* < 0.05), and infection with *H. suis* strain HS5cLPΔ*ggt* revealed significantly higher ASCT2 expression levels compared to WT *H. suis* infection (Figure 6B, *p* < 0.05). Similar results were observed in *H. pylori* infected mice, without being statistically significant (Figure 6, *p* > 0.05).

## Discussion

Although, in the present study, *H. suis* strain HS5cLPΔ*ggt* was shown to be able to colonize the stomach of mice at similar levels compared to WT *H. suis*, it induced significantly less overall inflammation in both corpus and antrum. This suggests that the *H. suis* GGT is involved in the induction and regulation of the inflammatory response, without being an essential factor for colonization. However, in Mongolian gerbils, *H. suis* strain HS5cLPΔ*ggt* was shown to induce only a slightly milder inflammatory response compared to the WT *H. suis* strain. This implies that, besides GGT, *H. suis* harbours other virulence factors or bacterial components, involved in the generation and modulation of the host immune response. In a previous study performed *in vitro*, lysate from HS5cLPΔ*ggt* indeed was shown to still have an effect on the proliferation and function of T lymphocytes, further suggesting the presence of hitherto unidentified factors in *H. suis* that can modulate the host immune and inflammatory response [20]. These factors remain to be investigated in the future.

Interestingly and in contrast to what we observed for *H. suis* lacking GGT, *H. pylori* strains SS1Δ*ggt* and PMSS1Δ*ggt* failed to persistently colonize the stomach of mice and gerbils, highlighting the different relative contributions of *H. pylori* GGT and *H. suis* GGT to the colonization ability in these rodent models. In any case, data from the current study as well as previous studies on the *H. pylori* GGT show that the *H. pylori* GGT confers a benefit to *H. pylori* in terms of its colonization capacity, at least in mice and gerbils, whereas the *H. suis* GGT mainly affects the inflammatory response evoked during *H. suis* infection without having a notable impact on the levels of bacterial colonization. Since *H. suis* lacks several other virulence determinants of *H. pylori,* such as VacA, the role of *H. suis* GGT in inducing or shaping the host immune response appears to be relatively important.

Our study reveals that *H. suis* infection induces a Th17 response in mice, without a significant upregulation of Th1 cytokines such as IFN-γ. This confirms the results of a previous study in which both Th1- and Th2- prone mouse strains were used [29]. However, the use of Mongolian gerbils in the present study demonstrated that *H. suis* infection can induce a marked upregulation of IFN-γ expression in this animal model, which is accompanied by a more pronounced gastritis compared to that seen in mice. For *H. pylori*, it has been demonstrated that infection induces the expression of IFN-γ in both mice and gerbils, which plays a pivotal role in promoting mucosal inflammation. This in turn contributes to more pronounced gastric mucosal damage [40]. Thus, the higher levels of IFN-γ expression in gerbils infected with *H. suis* most likely contribute to the more pronounced inflammation observed in this animal model compared to that in mice.

IL-10 is considered an important anti-inflammatory cytokine, which is mainly produced by regulatory T cells and dendritic cells [41], and this cytokine has been described to be upregulated in WT *H. suis* infected mice [29]. In the present study, we observed a similar expression pattern for IL-10 and Foxp3 in mice. This may indicate that the secretion of IL-10 mainly occurs through Tregs in the stomach, which needs to be confirmed in future studies. It may be postulated that the higher levels of IL-10 expression in HS5cLPΔ*ggt* infected mice are partially responsible for the attenuated inflammatory response, when compared to WT-infected animals. Previously published data from *in vitro* experiments have shown that *H. pylori* GGT suppresses IL-10 secretion by activated human CD4^+^ T cells [42], which is supported by our findings. The enzyme has, however, also been described to reprogram DC towards a tolerogenic phenotype, which was shown to depend upon increased secretion of IL-10 [43].

A pronounced upregulation of CXCL13 expression levels was observed in *H. suis*-infected animals, which was shown to be independent of the presence of *H. suis* GGT. Interestingly, a similar upregulation was completely absent in *H. pylori*-infected animals. Possibly, however, a longer experimental period (e.g. 12–18 months) may induce upregulation of CXCL13 expression in the stomach of these animals as well. CXCL13, also named B-cell-attracting chemokine-1 or B-lymphocyte chemoattractant, is a CXC subtype member of the chemokine superfamily [44], and it may play a pivotal role in various immune and inflammatory conditions as well as *H. pylori*-associated gastritis in humans [45,46]. It has been shown that the expression of CXCL13 is significantly upregulated in gastric MALT lymphoma in both humans [47] and mice [48]. The pronounced upregulation of CXCL13 as well as the presence of a clear proliferation of B-cells in germinal centers in the present study seem to be in line with the higher risk to develop gastric MALT lymphoma in humans infected with NHPH compared to *H. pylori* infected patients [5,49-51]. A recent report showed that the formation of gastric lymphoid follicles after challenge with gastric mucosal homogenate from a monkey harbouring *H. suis* was efficiently suppressed by the administration of anti-CXCL13 antibodies [14]. Taken together, this shows that CXCL13 might be one of the key cytokines involved in the development of gastric MALT lymphoma associated with *H. suis* infection.

In previous experiments we have shown that *H. suis* GGT inhibits the proliferation of lymphocytes *in vitro* through the interaction with glutamine [20]. This seems contradictory to the results of the present *in vivo* study showing that animals infected with *H. suis* strain HS5cLPΔ*ggt* exhibited a lower lymphocytic infiltration rate in the gastric mucosa. Besides lymphocytes, however, *H. suis* and its GGT also target gastric mucosal epithelial cells [19]. The uncontrolled loss of epithelial cells by cell death, e.g. necrosis, also triggers the influx of inflammatory cells, in turn promoting the further development of inflammation. In line with some of our previous studies [4], *H. suis* infection indeed affected the function of gastric acid secreting parietal cells, as shown by the decreased expression levels of *Atp4a* and *Atp4b*, and the mutant work demonstrated that *H. suis* GGT indeed plays a role. In addition, the present study indicates that the epithelial (hyper)proliferation observed in WT *H. suis* infected mice is more pronounced than in HS5cLPΔ*ggt* infected mice. This suggests that *H. suis* lacking GGT causes less damage to the epithelium compared to WT bacteria. Probably, this also has an implication on the subsequent development of inflammation in the presence of a more or less damaged epithelium. However, it remains to be determined whether the impact of the *H. suis* GGT on the health of gastric epithelial cells is stronger compared to its direct effects on lymphocytes residing in the deeper tissue layers, including the inhibitory effect on their proliferation.

As mentioned above, infection with *H. suis* strain HS5cLP in mice induced a clear downregulation of *Atp4a* and *Atp4b* expression levels in the stomach at 9 weeks and 6 months pi, and such an effect was not observed in the HS5cLPΔ*ggt* infected animals, showing that *H. suis* GGT contributes to alterations in gastric acid secretion by parietal cells. Previous reports have shown that *H. suis* is often observed near or inside the canaliculi of parietal cells in the stomach of mice, and colonization of *H. suis* is also closely linked with necrosis of parietal cells in mice and Mongolian gerbils [4]. Besides the direct effect of *H. suis* GGT on the acid secretion by parietal cells, altered expression levels of IL-1β may also affect the acid production through multiple pathways [52,53], including a decreased histamine release from enterochromaffin-like cells [54]. The impaired gastric acid secretion and subsequent development of mucous metaplasia observed in the present study, may lead to the development of gastric atrophy, hypochlorhydria and gastric cancer [55,56].

For the first time, we were able to show an effect of *H. suis* GGT on the glutamine metabolism of gastric epithelial cells. This amino acid, targeted by the enzymatic activity of *H. suis* GGT [20], is a major fuel for rapidly dividing cells, including enterocytes, macrophages and lymphocytes [57,58]. It is supportive in improving digestion, absorption, and retention of nutrients through affecting tissue anabolism, stress, and immunity, and it also plays an important role in animal nutrition and health. WT *H. suis* infection was shown to cause a significant downregulation of ASCT2 mRNA in mice, while HS5cLPΔ*ggt* did not show this effect. This suggests that glutamine depletion catalysed by GGT activity at the level of the gastric mucosa resulted in the downregulation of glutamine transporter ASCT2. ASCT2 is a Na^+^-dependent, broad-scope neutral amino acid transporter [59,60], which is essential for glutamine uptake by fast growing epithelial cells and tumor cells [39,61,62], and ASCT2 expression levels depend on glutamine availability [63].

In summary, our data show that *H. suis* GGT is not an essential factor for colonization in mice and gerbils, whereas it is involved in the induction of an inflammatory response. This differs to what has been described for the *H. pylori* GGT. In addition, we demonstrated that *H. suis* infection causes a considerable increase of IFN-γ expression levels in Mongolian gerbils, which differs from the situation in mice, where *H. suis* infection is not accompanied by increased expression of this Th1 signature cytokine. This Th1 response was shown to be attenuated in the absence of *H. suis* GGT. CXCL13 expression levels were shown to be upregulated during *H. suis* infection, in contrast to what we observed for *H. pylori* infection, and this was shown not to depend on the presence of *H. suis* GGT. WT *H. suis* infection was shown to suppress expression levels of *Atp4a* and *Atp4b*, involved in gastric acid secretion, and to suppress expression levels of the glutamine transporter ASCT2. These effects on the gastric epithelium were clearly related to the presence of *H. suis* GGT.

## Additional files

Additional file 1:
**H&E staining of the stomach section from a** ***Helicobacter suis*** **infected Mongolian gerbil.** The vast majority of the antrum of the stomach from this WT *H. suis-*infected animal was densely infiltrated with inflammatory cells, fused lymphoid aggregates and lymphoid follicles. Original magnification: 25 ×. Additional file 2:
**Proliferation of B cells in germinal centers.** Representative micrographs of a Ki67 staining of the stomach from a WT *H. suis* infected (A) and *H. suis*Δ*ggt* infected gerbil (B) are shown. Proliferating germinal centers were observed in animals from both groups, but mainly in WT *H. suis* infected animals. WT: wild-type. Original magnification: 50× and 200 ×. Additional file 3:
**Immunohistochemical staining of the hydrogen potassium ATPase of parietal cells in the stomach mucosa of Mongolian gerbils.** Moderate numbers of parietal cells (brown) are present at the transition zone between the corpus and antrum of the stomach of control Mongolian gerbils (A). A clear loss of parietal cells is observed in the transition zone between the corpus and antrum of the stomach from Mongolian gerbils infected with WT *H. suis* strain HS5cLP (B) or *H. suis* strain HS5cLPΔ*ggt* (C) at 9 weeks post inoculation. No clear change of parietal cell numbers is seen in the transition zone between the corpus and antrum of the stomach from Mongolian gerbils infected with WT *H. pylori* PMSS1 (D) or *H. pylori* PMSS1Δ*ggt* (E) at 9 weeks post inoculation. WT: wild-type. Original magnification: 100 ×. Additional file 4:
**Determination of mucous metaplasia in the stomach from** ***Helicobacter*** **-infected mice.** An AB/PAS staining was applied to determine the presence of pseudopyloric metaplasia (arrows) in the stomachs of control mice (A), WT *H. suis* infected mice (B), *H. suis*Δ*ggt* infected mice (C), WT *H. pylori* infected mice (D), and *H. pylori*Δ*ggt* infected mice (E) at 6 months post infection. WT: wild-type; AB/PAS: alcial blue-periodic acid-Schiff stain. Original magnification: 100 ×.
