# Supplementary figures and images for: Role of γ-glutamyltranspeptidase in the pathogenesis of Helicobacter suis and Helicobacter pylori infections
Source: Vet Res. 2015 Mar 13;46:31. doi: 10.1186/s13567-015-0163-6 (PMC4357089; doi:10.1186/s13567-015-0163-6)

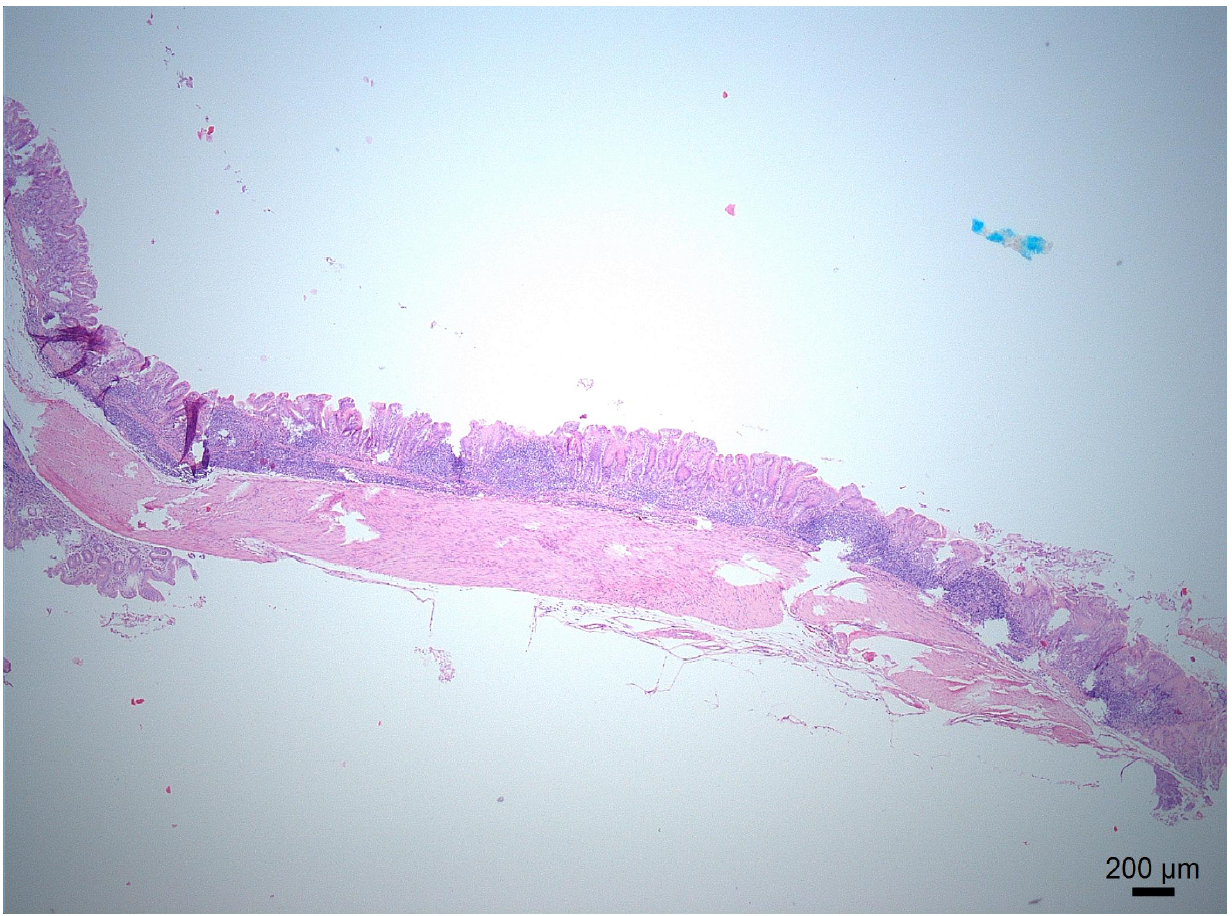

Supplement: Additional file 1: — H&E staining of the stomach section from a Helicobacter suis infected Mongolian gerbil. The vast majority of the antrum of the stomach from this WT H. suis-infected animal was densely infiltrated with inflammatory cells, fused lymphoid aggregates and lymphoid follicles. Original magnification: 25 ×. [file 13567_2015_163_MOESM1_ESM.tif]

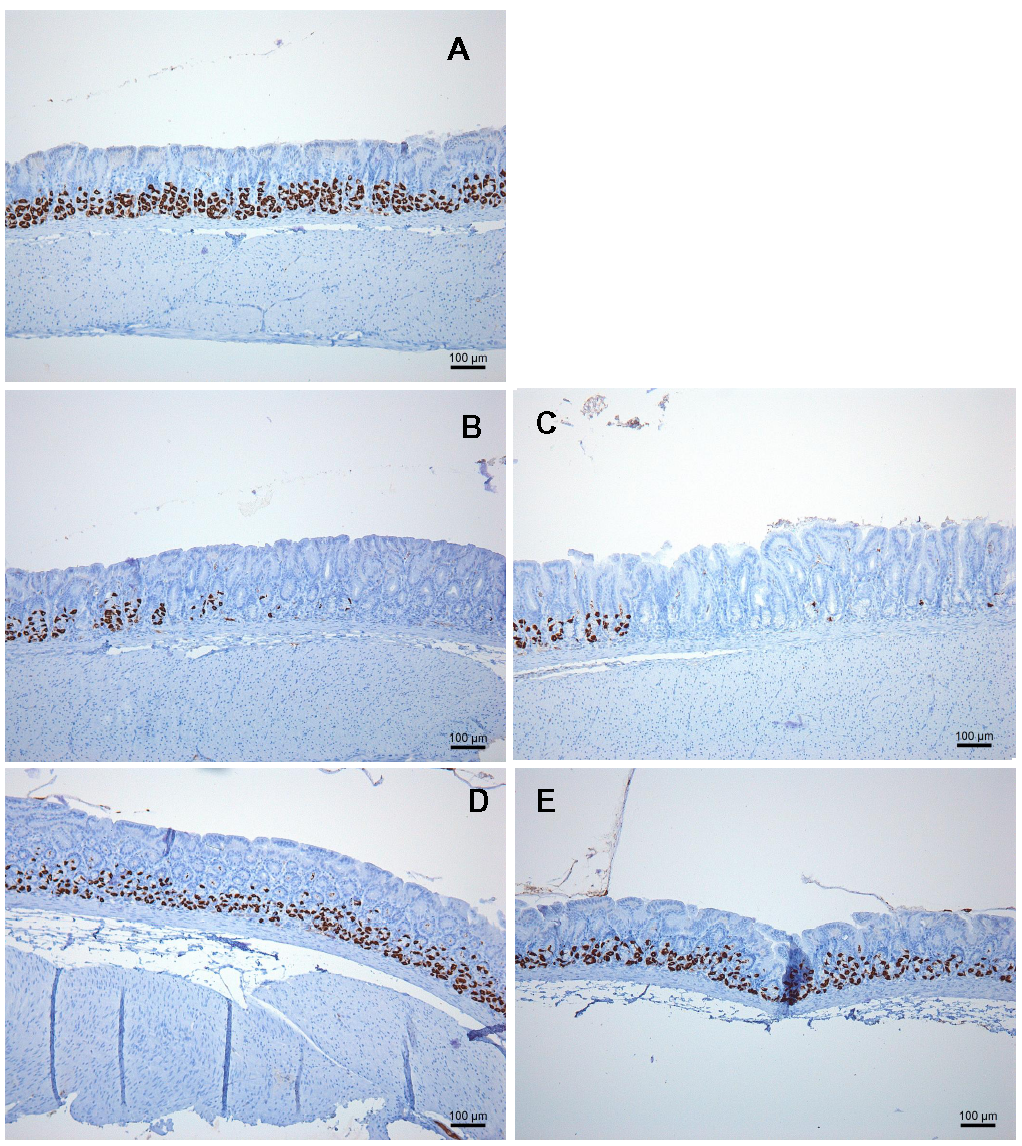

Supplement: Additional file 2: — Proliferation of B cells in germinal centers. Representative micrographs of a Ki67 staining of the stomach from a WT H. suis infected (A) and H. suisΔggt infected gerbil (B) are shown. Proliferating germinal centers were observed in animals from both groups, but mainly in WT H. suis infected animals. WT: wild-type. Original magnification: 50× and 200 ×. [file 13567_2015_163_MOESM2_ESM.tif]

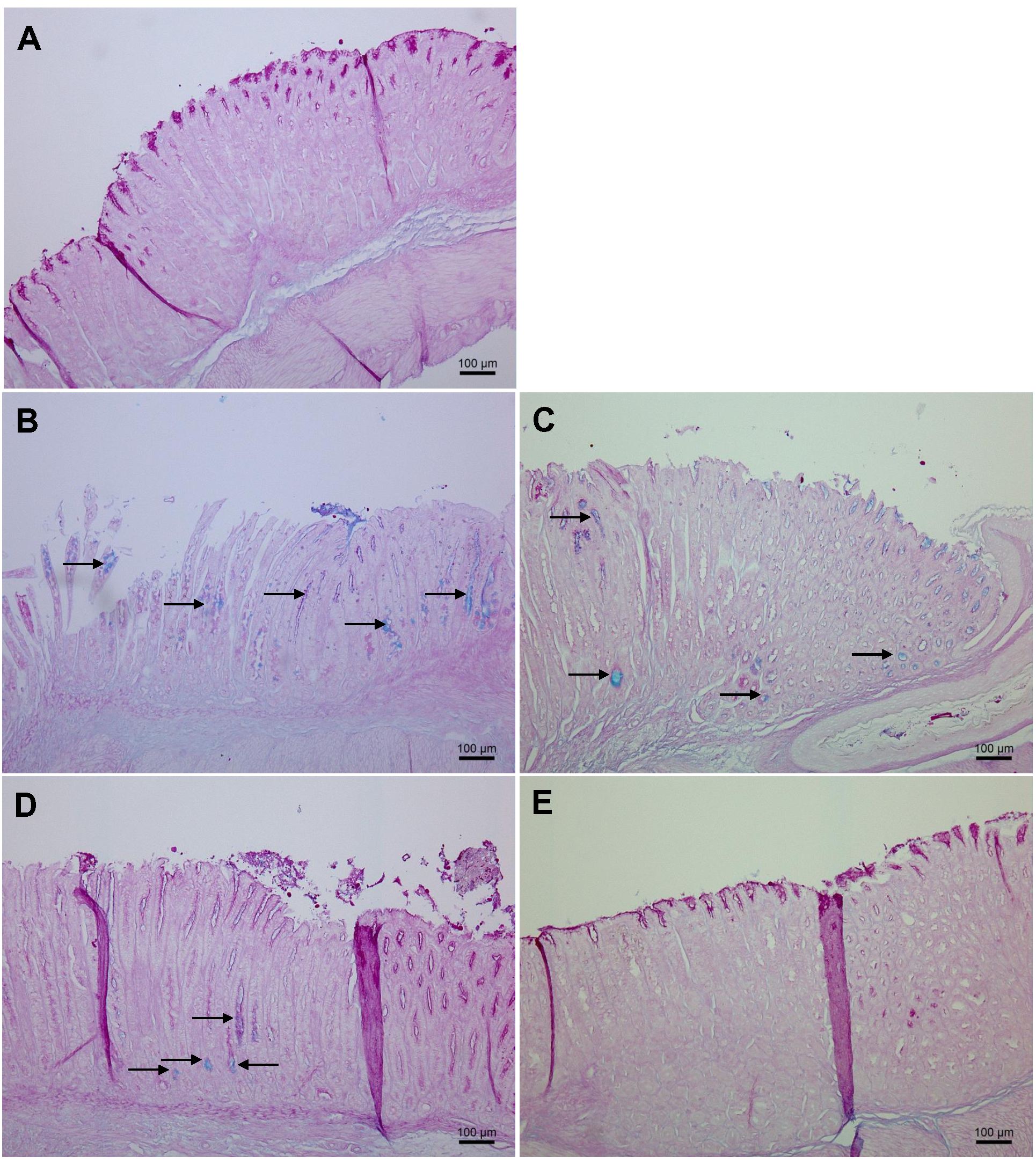

Supplement: Additional file 3: — Immunohistochemical staining of the hydrogen potassium ATPase of parietal cells in the stomach mucosa of Mongolian gerbils. Moderate numbers of parietal cells (brown) are present at the transition zone between the corpus and antrum of the stomach of control Mongolian gerbils (A). A clear loss of parietal cells is observed in the transition zone between the corpus and antrum of the stomach from Mongolian gerbils infected with WT H. suis strain HS5cLP (B) or H. suis strain HS5cLPΔggt (C) at 9 weeks post inoculation. No clear change of parietal cell numbers is seen in the transition zone between the corpus and antrum of the stomach from Mongolian gerbils infected with WT H. pylori PMSS1 (D) or H. pylori PMSS1Δggt (E) at 9 weeks post inoculation. WT: wild-type. Original magnification: 100 ×. [file 13567_2015_163_MOESM3_ESM.tif]

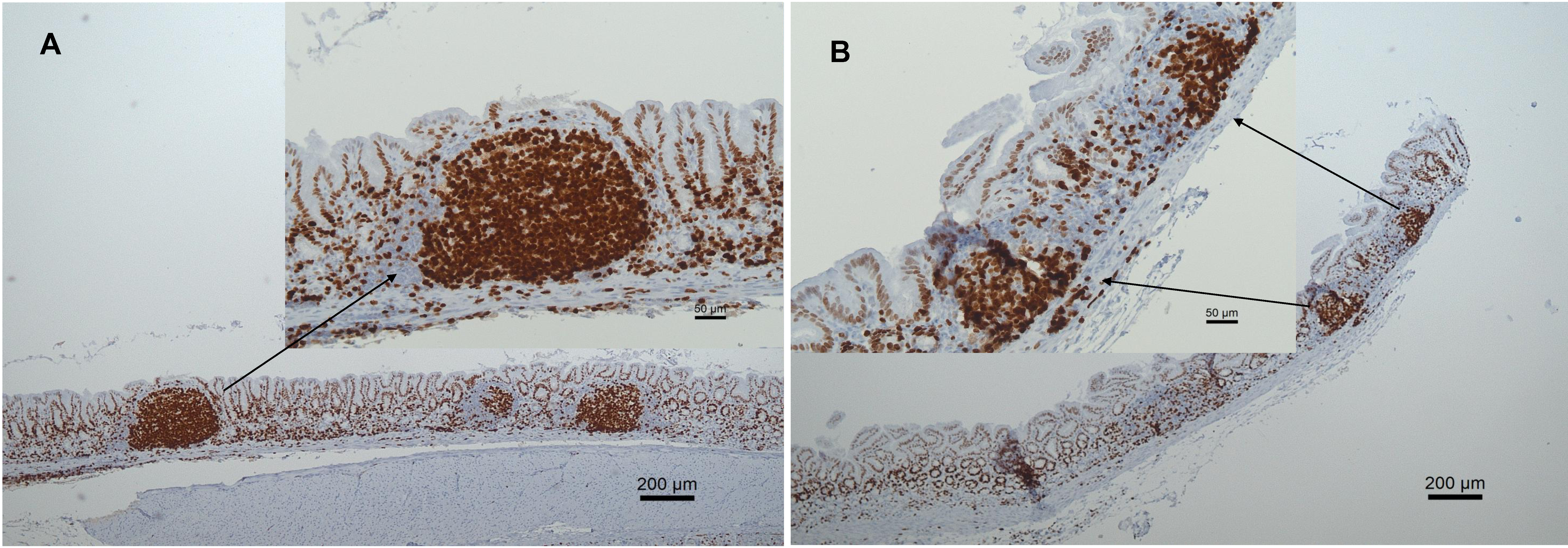

Supplement: Additional file 4: — Determination of mucous metaplasia in the stomach from Helicobacter -infected mice. An AB/PAS staining was applied to determine the presence of pseudopyloric metaplasia (arrows) in the stomachs of control mice (A), WT H. suis infected mice (B), H. suisΔggt infected mice (C), WT H. pylori infected mice (D), and H. pyloriΔggt infected mice (E) at 6 months post infection. WT: wild-type; AB/PAS: alcial blue-periodic acid-Schiff stain. Original magnification: 100 ×. [file 13567_2015_163_MOESM4_ESM.tif]
